# Supplementary material for: Genetic Characterization, Transmission Pattern and Health Risk Analysis of Intestinal Colonization ESBL-Producing Escherichia coli in Vegetable Farming Population
Source: Microorganisms. 2024 Dec 20;12(12):2646. doi: 10.3390/microorganisms12122646 (PMC11727906; doi:10.3390/microorganisms12122646)
Supplement: Supplementary file 1 [file microorganisms-12-02646-s001.zip › Supplementary materials/Supplementary materials.pdf]

Table S1. Resistance spectrum of 45 intestinal colonized ESBL-Ec isolates to 12 common antibiotics

Isolate	Minimum inhibitory concentration(mg/L)											
	TZP	IMM	MEM	CIP	CAZ	FOS	AMK	CTX	TE	U	PB	TGC
F2	16/4	0.5	0.03125	>32	8	64	2	32	>64	>256	2	0.5
F3	32/4	0.5	0.03125	32	16	>512	4	32	>64	64	2	0.25
F4	16/4	0.5	0.03125	>32	16	>512	2	64	>64	>256	2	0.25
F5	16/4	0.5	0.03125	1	8	>512	4	>64	>64	>256	2	0.25
F7	16/4	0.5	0.0625	>32	>64	>512	4	>64	>64	64	2	0.125
F8	16/4	0.5	0.03125	2	16	>512	2	64	64	32	1	0.125
F9	32/4	0.5	0.03125	16	16	>512	4	64	>64	64	2	0.125
F10	64/4	1	0.03125	32	16	>512	4	64	>64	>256	1	0.25
F11	32/4	0.5	0.03125	>32	64	>512	4	64	>64	>256	2	0.125
F13	32/4	1	0.03125	32	16	>512	2	64	>64	>256	1	0.125
F14	32/4	1	0.03125	32	>64	>512	4	32	>64	64	2	0.125
F15	16/4	1	0.03125	2	16	>512	2	64	>64	64	2	0.125
F16	32/4	0.5	0.03125	32	32	>512	4	>64	>64	>256	2	0.25
F17	32/4	1	0.03125	1	8	256	2	32	>64	32	1	0.25
F19	32/4	8	0.0625	1	>64	256	8	64	>64	32	1	0.25
F21	32/4	0.5	0.03125	>32	16	>512	4	>64	>64	>256	1	0.25
F23	32/4	0.5	0.03125	>32	8	>512	2	32	>64	>256	0.5	0.25
F25	8/4	0.5	0.03125	1	1	128	4	16	>64	64	2	0.125
F26	256/4	1	0.25	>32	>64	>512	4	>64	>64	>256	2	0.25
F27	16/4	0.25	0.03125	1	8	>512	2	32	>64	>256	2	0.25
F28	16/4	0.25	0.03125	0.5	16	64	2	32	8	64	2	0.25
F30	16/4	0.5	0.03125	2	8	64	4	32	16	>256	1	0.5
F35	16/4	1	0.03125	2	16	64	1	64	32	64	1	0.25
F37	16/4	1	0.03125	>32	16	>512	4	64	>64	>256	1	0.25
F40	16/4	0.5	0.03125	>32	>64	>512	1	32	>64	>256	0.5	0.25
F41	64/4	0.5	0.03125	>32	16	>512	8	64	>64	>256	0.5	0.25
F42	16/4	1	0.03125	>32	4	>512	4	32	>64	>256	2	0.25
F43	32/4	1	0.03125	32	2	16	4	32	>64	>256	2	0.25
F44	256/4	0.25	0.25	1	>64	>512	2	64	>64	>256	2	0.5
F46	16/4	0.25	0.03125	0.5	1	64	4	16	>64	64	2	0.25
F47	16/4	0.5	0.03125	1	2	>512	4	16	8	64	1	0.25
F49	16/4	0.5	0.03125	32	8	>512	4	32	>64	>256	1	0.5
F52	32/4	1	0.03125	>32	>64	256	1	>64	>64	4	0.5	0.5
F53	32/4	1	0.03125	0.5	4	>512	2	4	>64	>256	0.5	0.5
F54	32/4	1	0.03125	>32	16	>512	8	32	>64	>256	2	0.5
F55	16/4	0.5	0.03125	>32	2	>512	4	16	>64	>256	1	0.25
F57	16/4	1	0.03125	>32	4	>512	4	32	>64	64	1	0.5
F58	16/4	0.25	0.03125	>32	16	>512	2	32	>64	4	1	0.5
F59	16/4	0.5	0.03125	>32	1	>512	4	32	>64	>256	0.25	0.25
F61	32/4	0.5	0.03125	4	4	>512	2	32	>64	>256	1	0.25
F62	16/4	0.5	0.03125	1	16	>512	2	32	>64	64	0.5	0.25
F63	8/4	0.5	0.03125	1	2	>512	4	16	>64	64	1	0.25
F64	64/4	0.5	0.03125	8	>64	>512	4	32	>64	>256	1	0.5
F65	16/4	0.25	0.03125	>32	16	>512	4	32	>64	>256	1	0.5
F66	16/4	1	0.03125	>32	4	>512	4	32	>64	>256	1	0.25

Red: resistant（R）, yellow: intermediary（I）, green: sensitive（S）

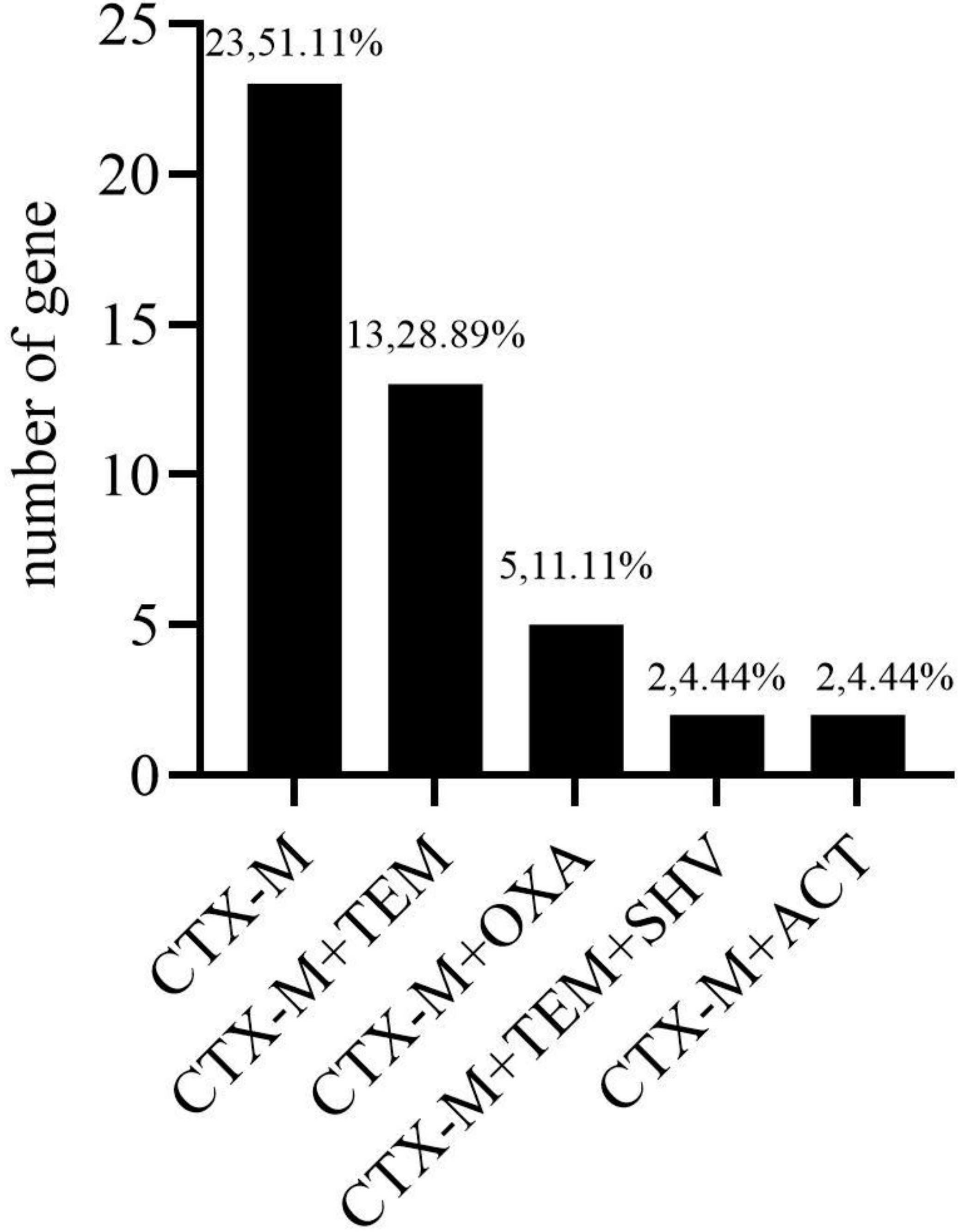

Figure. S1 Combination of β-lactam resistance genes

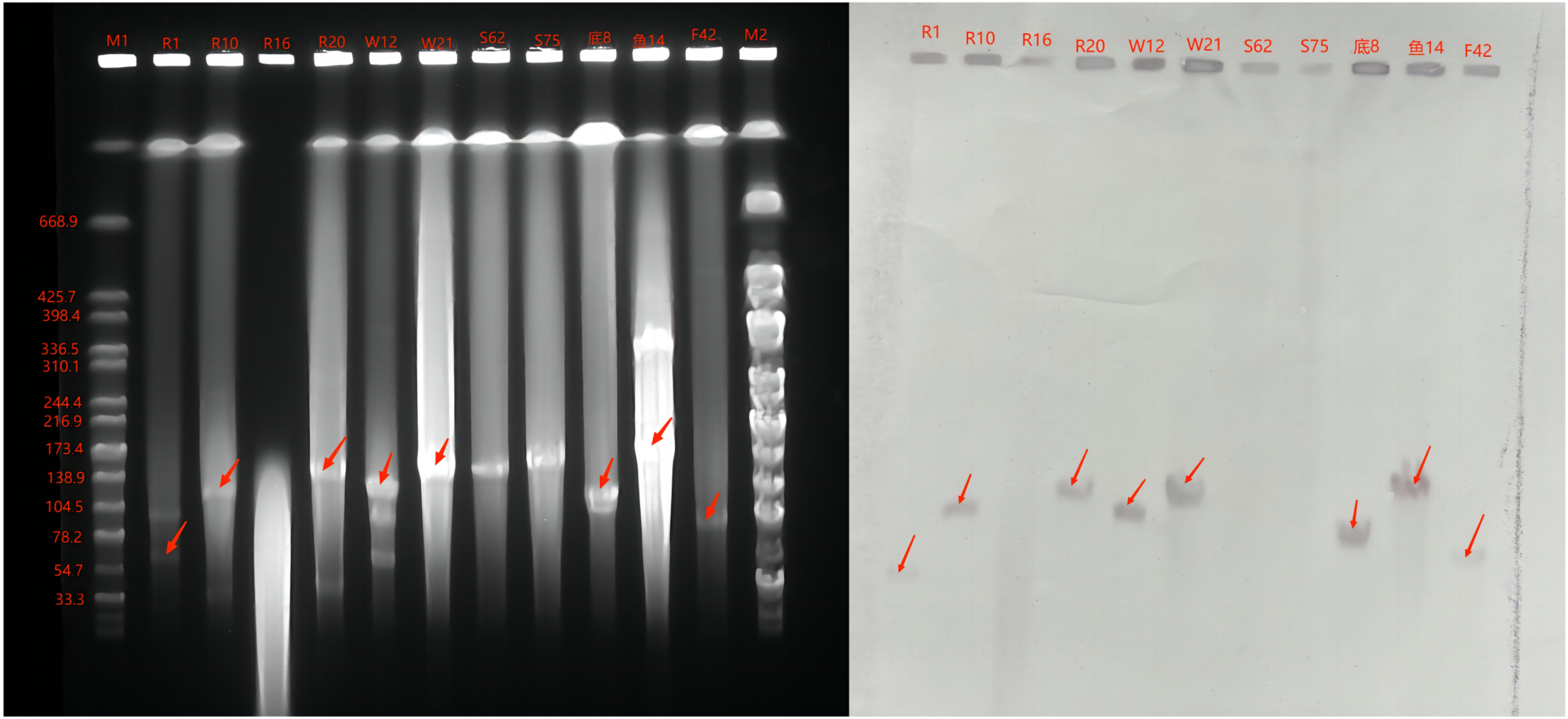

Figure.S2(A)

S1-PFGE and Southern blot hybridization results, with red arrows marking the locations of the transferable plasmids where the resistance genes are located.

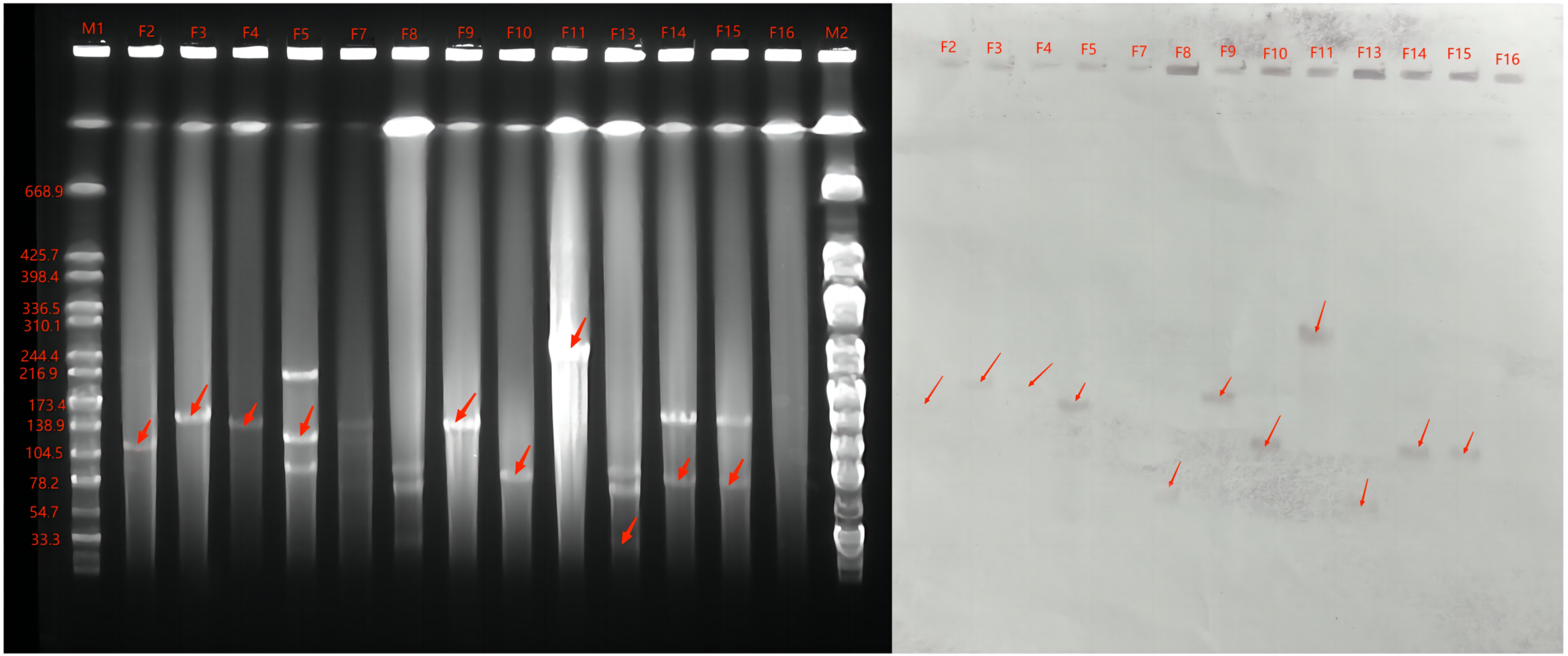
M1
F2
F3
F4
F5
F7
F8
F9
F10
F11
F13
F14
F15
F16
M2
668.9
425.7
398.4
336.5
310.1
244.4
216.9
173.4
138.9
104.5
78.2
54.7
33.3
F2
F3
F4
F5
F7
F8
F9
F10
F11
F13
F14
F15
F16

Figure.S2(B)

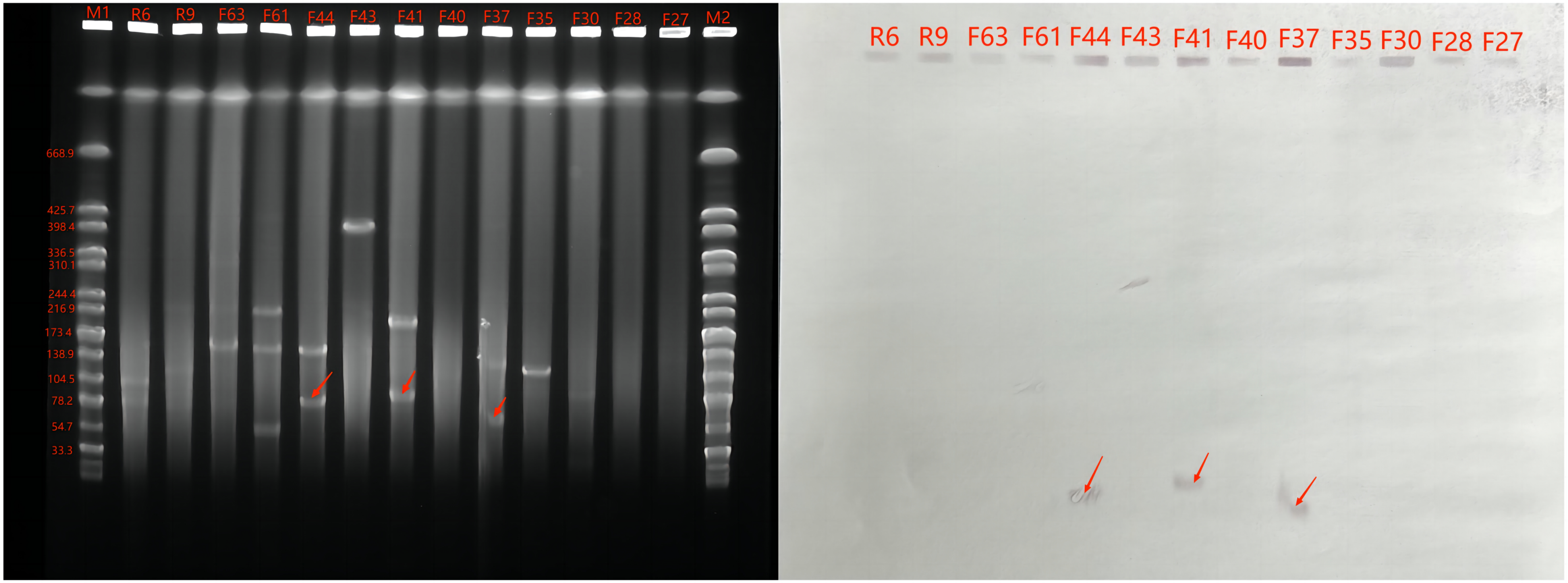
M1
R6
R9
F63
F61
F44
F43
F41
F40
F37
F35
F30
F28
F27
M2
668.9
425.7
398.4
336.5
310.1
244.4
216.9
173.4
138.9
104.5
78.2
54.7
33.3
R6
R9
F63
F61
F44
F43
F41
F40
F37
F35
F30
F28
F27

Figure.S2(C)

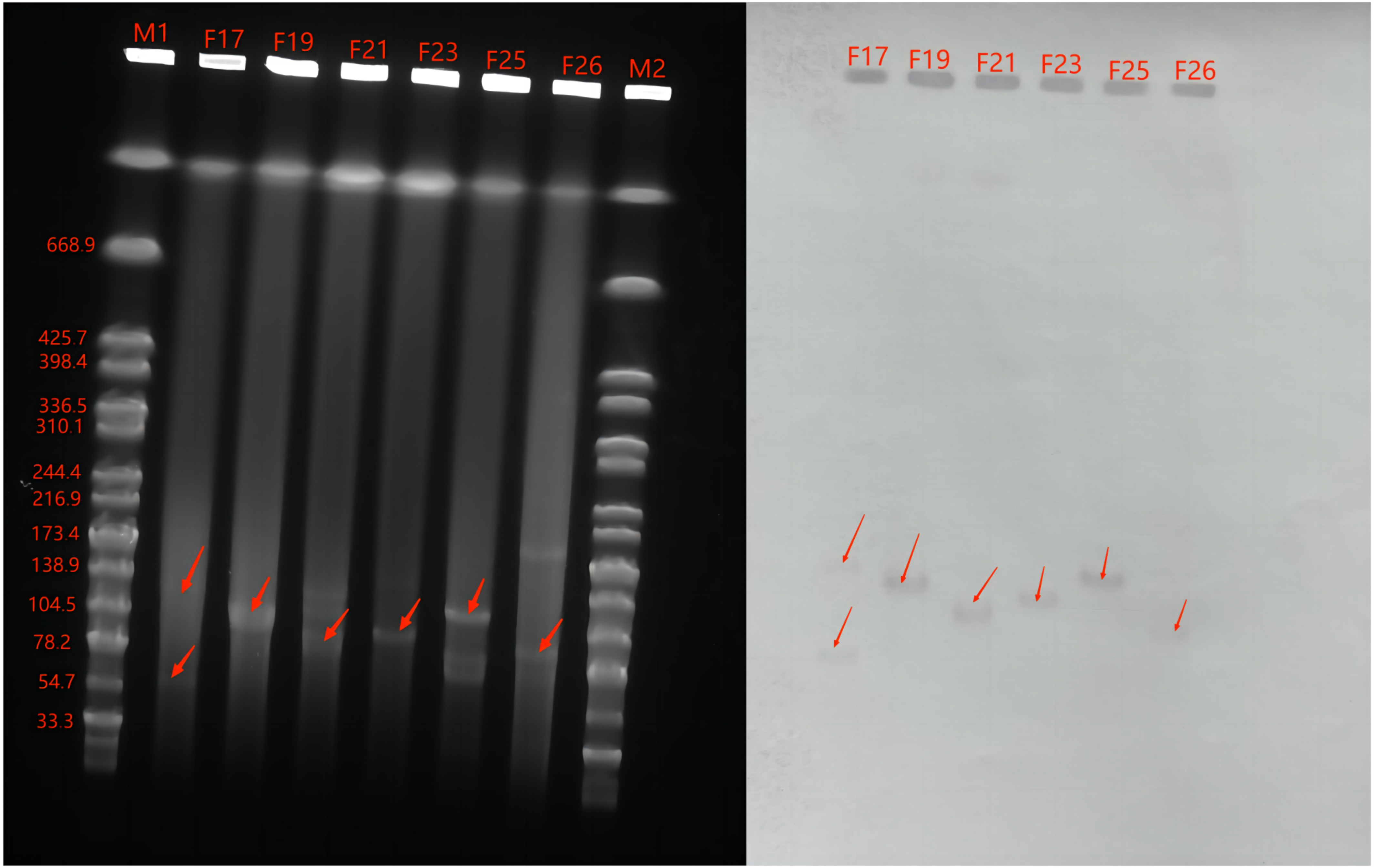
M1
F17
F19
F21
F23
F25
F26
M2
668.9
425.7
398.4
336.5
310.1
244.4
216.9
173.4
138.9
104.5
78.2
54.7
33.3
F17
F19
F21
F23
F25
F26

Figure.S2(D)

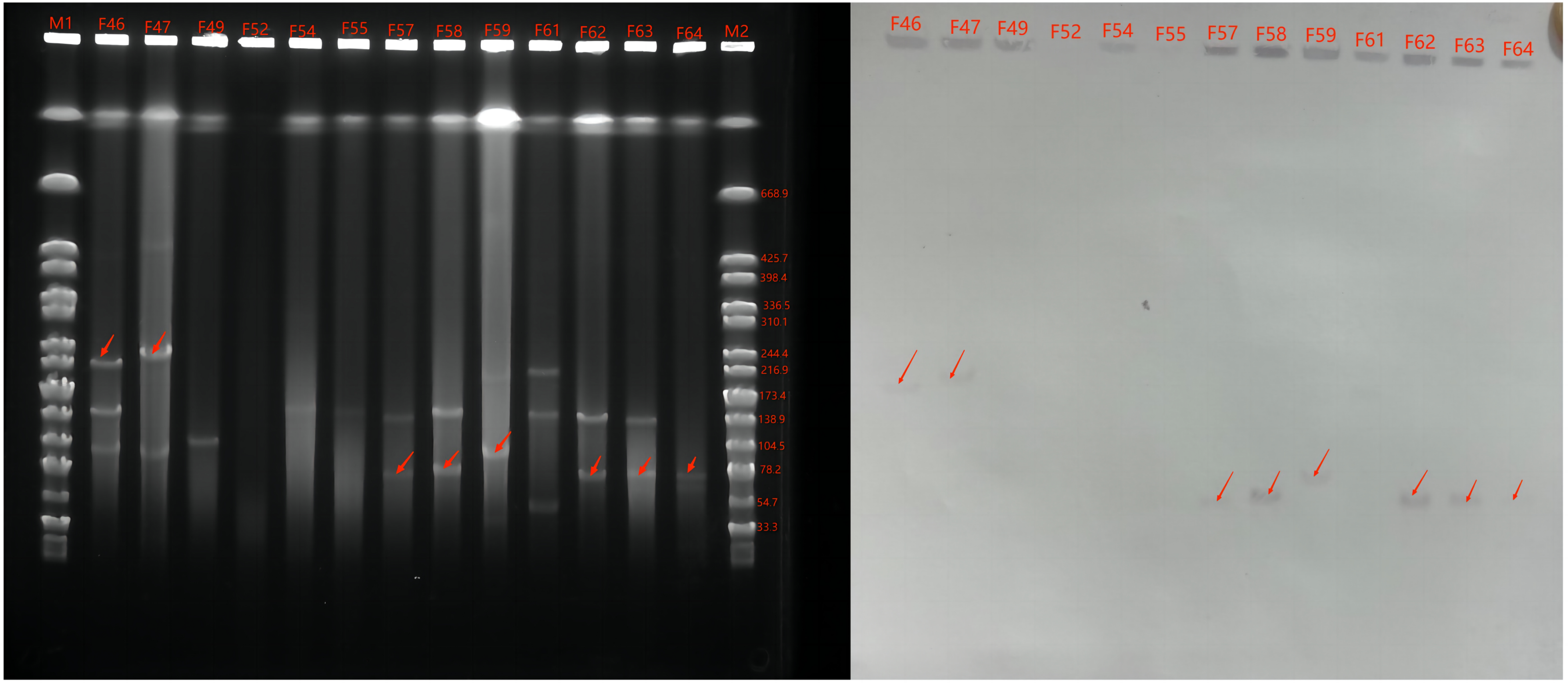
M1
F46
F47
F49
F52
F54
F55
F57
F58
F59
F61
F62
F63
F64
M2
668.9
425.7
398.4
336.5
310.1
244.4
216.9
173.4
138.9
104.5
78.2
54.7
33.3
F46
F47
F49
F52
F54
F55
F57
F58
F59
F61
F62
F63
F64

Figure.S2(E)

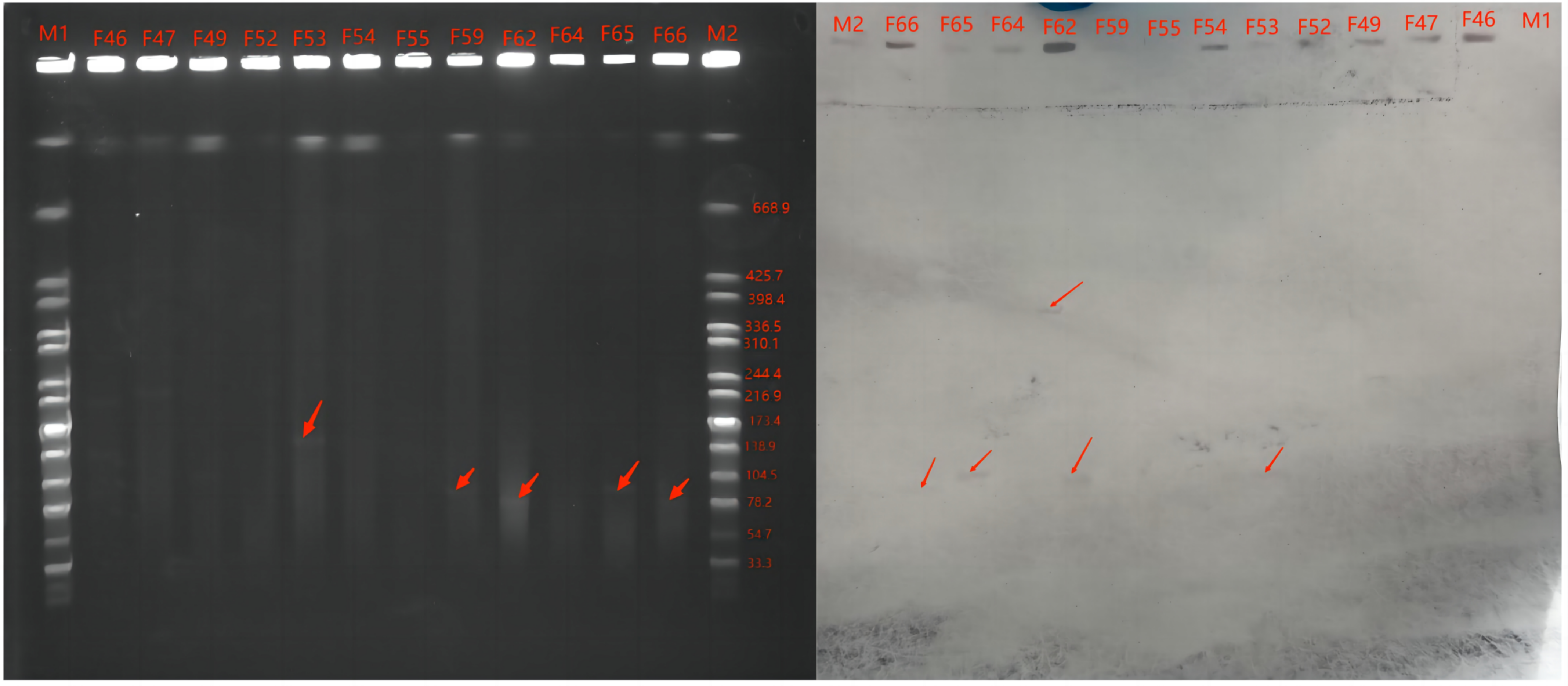
M1
F46
F47
F49
F52
F53
F54
F55
F59
F62
F64
F65
F66
M2
668.9
425.7
398.4
336.5
310.1
244.4
216.9
173.4
138.9
104.5
78.2
54.7
33.3
M2
F66
F65
F64
F62
F59
F55
F54
F53
F52
F49
F47
F46
M1

Figure.S2(F)

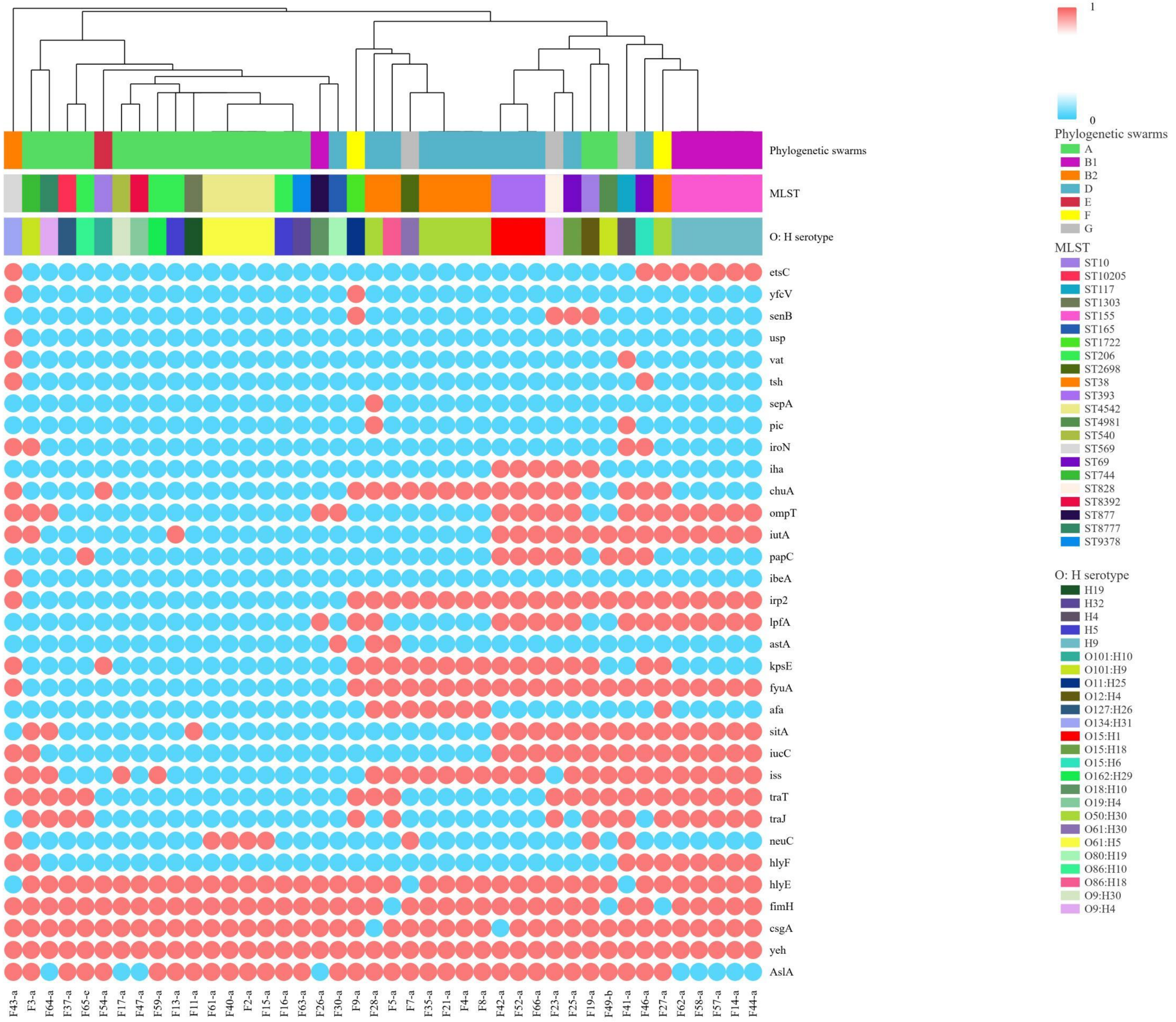

Figure.S3 Clustering of phylogenetic groups, MLST types and serotypes to analyze the virulence genes carried by intestinal colonized ESBL-Ec isolates (red is carrying the virulence gene, blue is not carrying the virulence gene)

Table S2.Detailed information of ESBL-Ec isolates from different time and sources in Shandong Province, China

Isolate number	Source	Year	Host	Isolation source
GCA_001893765.1	animal breeding area	2015	Homo sapiens	fecal swabs from commercial chicken farm
GCA_001894505.1	animal breeding area	2015	Homo sapiens	fecal swabs from commercial chicken farm
GCA_001894545.1	animal breeding area	2015	Homo sapiens	fecal swabs from commercial chicken farm
GCA_002967865.3	clinical	2014	Homo sapiens	blood
GCA_002967955.2	clinical	2014	Homo sapiens	urine
GCA_003111785.1	clinical	2016	Homo sapiens	feces
GCA_003112045.1	clinical	2016	Homo sapiens	feces
GCA_013039865.1	animal breeding area	2016	swine	Pig Lung
GCA_013039905.1	animal breeding area	2016	Broiler	Chicken Lung
GCA_013039925.1	animal breeding area	2016	Broiler	Chicken Lung
GCA_013039945.1	animal breeding area	2016	Broiler	Chicken Liver
GCA_013039845.1	animal breeding area	2016	Broiler	Chicken Lung
GCA_013039765.1	animal breeding area	2016	Broiler	Chicken faeces
GCA_013039875.1	animal breeding area	2016	Broiler	Chicken faeces
GCA_013039805.1	animal breeding area	2016	Broiler	Chicken Faeces
GCA_013039665.1	animal breeding area	2016	Broiler	Chicken Faeces
GCA_013039725.1	animal breeding area	2016	Broiler	Chicken Faeces
GCA_013038985.1	animal breeding area	2016	Broiler	Chicken Lung
GCA_013038805.1	animal breeding area	2016	Broiler	Chicken Liver
GCA_013039065.1	animal breeding area	2016	Broiler	Chicken Liver
GCA_013038745.1	animal breeding area	2016	Broiler	Chicken Feces
GCA_013039405.1	animal breeding area	2016	Broiler	Chicken Feces
GCA_013039025.1	animal breeding area	2016	Broiler	Chicken feces
GCA_013039155.1	animal breeding area	2016	Broiler	Chicken Faeces
GCA_013039605.1	animal breeding area	2016	Broiler	Chicken Faeces
GCA_013039565.1	animal breeding area	2016	Broiler	Chicken faeces
GCA_013039525.1	animal breeding area	2016	Broiler	Chicken Faeces
GCA_013039545.1	animal breeding area	2016	Broiler	Chicken Faeces
GCA_013039485.1	animal breeding area	2016	Broiler	Chicken Faeces
GCA_013039505.1	animal breeding area	2016	swine	Pig Liver
GCA_013039465.1	clinical	2013	Homo sapiens	Patient Abdominal fluid
GCA_013039445.1	clinical	2013	Homo sapiens	Patient Blood
GCA_013039085.1	clinical	2016	Homo sapiens	Patient Urine
GCA_013038945.1	clinical	2016	Homo sapiens	Patient Urine
GCA_013038485.1	animal breeding area	2016	swine	sick pig lung
GCA_013038465.1	animal breeding area	2016	swine	Pig anus swab
GCA_013038445.1	animal breeding area	2016	swine	Pig anus swab
GCA_013038385.1	animal breeding area	2016	Broiler	Chicken manure
GCA_013038425.1	animal breeding area	2016	Broiler	sick animal
GCA_013038245.1	animal breeding area	2016	Broiler	sick animal
GCA_013038525.1	animal breeding area	2016	Broiler	sick animal
GCA_013038165.1	animal breeding area	2016	Broiler	sick animal
GCA_013038345.1	animal breeding area	2013	Broiler	Chicken Anal swab
GCA_013038195.1	animal breeding area	2013	Broiler	Chicken Anal swab
GCA_013038125.1	animal breeding area	2013	Broiler	Chicken Anal swab
GCA_013038145.1	animal breeding area	2013	Broiler	Chicken Anal swab
GCA_013038105.1	animal breeding area	2013	Broiler	Chicken Anal swab
GCA_013038065.1	animal breeding area	2013	Broiler	Chicken Liver
GCA_013037945.1	clinical	2016	Homo sapiens	Patient Sputum
GCA_009393715.1	clinical	2015	Homo sapiens	fecal sample
GCA_009393735.1	clinical	2015	Homo sapiens	fecal sample
GCA_009393745.1	clinical	2015	Homo sapiens	fecal sample
GCA_009393765.1	clinical	2015	Homo sapiens	fecal sample

GCA_009393835.1	clinical	2015	Homo sapiens	fecal sample
GCA_009394465.1	clinical	2015	Homo sapiens	fecal sample
GCA_009394485.1	clinical	2015	Homo sapiens	fecal sample
GCA_009394525.1	clinical	2015	Homo sapiens	fecal sample
GCA_009394575.1	clinical	2015	Homo sapiens	fecal sample
GCA_009394595.1	clinical	2015	Homo sapiens	fecal sample
GCA_009394585.1	clinical	2015	Homo sapiens	fecal sample
GCA_009394625.1	clinical	2015	Homo sapiens	fecal sample
GCA_015687295.1	clinical	2018	Homo sapiens	feces
GCA_015687305.1	clinical	2018	Homo sapiens	feces
GCA_015687355.1	clinical	2018	Homo sapiens	feces
GCA_015687375.1	clinical	2018	Homo sapiens	feces
GCA_015687385.1	clinical	2018	Homo sapiens	feces
GCA_015687395.1	clinical	2018	Homo sapiens	feces
GCA_015687435.1	clinical	2018	Homo sapiens	feces
GCA_015687455.1	clinical	2018	Homo sapiens	feces
GCA_015731735.1	clinical	2018	Homo sapiens	feces
GCA_015843075.1	clinical	2018	Homo sapiens	Urine sample
GCA_018295485.1	clinical	2012	Homo sapiens	feces
GCA_018295345.1	clinical	2012	Homo sapiens	feces
GCA_018283795.1	clinical	2012	Homo sapiens	feces
GCA_018295285.1	clinical	2012	Homo sapiens	feces
GCA_018295305.1	clinical	2012	Homo sapiens	feces
GCA_018295265.1	clinical	2012	Homo sapiens	feces
GCA_018295245.1	clinical	2012	Homo sapiens	feces
GCA_018295195.1	clinical	2012	Homo sapiens	feces
GCA_018295225.1	clinical	2012	Homo sapiens	feces
GCA_018295185.1	clinical	2012	Homo sapiens	feces
GCA_018295165.1	clinical	2012	Homo sapiens	feces
GCA_018295145.1	clinical	2012	Homo sapiens	feces
GCA_018295125.1	clinical	2012	Homo sapiens	feces
GCA_018295105.1	clinical	2012	Homo sapiens	feces
GCA_018295085.1	clinical	2012	Homo sapiens	feces
GCA_018295045.1	clinical	2012	Homo sapiens	feces
GCA_018295065.1	clinical	2012	Homo sapiens	feces
GCA_018283835.1	clinical	2012	Homo sapiens	feces
GCA_018295025.1	clinical	2012	Homo sapiens	feces
GCA_018295005.1	clinical	2012	Homo sapiens	feces
GCA_018294985.1	clinical	2012	Homo sapiens	feces
GCA_018294965.1	clinical	2012	Homo sapiens	feces
GCA_018294945.1	clinical	2012	Homo sapiens	feces
GCA_018294905.1	clinical	2012	Homo sapiens	feces
GCA_018294925.1	clinical	2012	Homo sapiens	feces
GCA_018862035.1	animal breeding area	2019	duck	Farm
GCA_020168475.1	clinical	2018	Homo sapiens	patient with infection
GCA_020168515.1	clinical	2019	Homo sapiens	patient with infection
GCA_020168535.1	clinical	2019	Homo sapiens	patient with infection
GCA_020168555.1	clinical	2019	Homo sapiens	patient with infection
GCA_020168575.1	clinical	2019	Homo sapiens	patient with infection
GCA_020421445.1	animal breeding area	2018	Mink	lung
GCA_020421525.1	animal breeding area	2019	Mink	lung
GCA_020421565.1	animal breeding area	2019	Mink	lung
GCA_020421595.1	animal breeding area	2017	Mink	lung
GCA_020421625.1	animal breeding area	2017	Mink	lung
GCA_020421645.1	animal breeding area	2018	Mink	lung
GCA_020421665.1	animal breeding area	2019	Mink	lung

GCA_020421655.1	animal breeding area	2018	Mink	lung
GCA_020421675.1	animal breeding area	2018	Mink	lung
GCA_020421725.1	animal breeding area	2017	Mink	lung
GCA_020421765.1	animal breeding area	2017	Mink	lung
GCA_021488875.1	clinical	2017	Homo sapiens	feces
GCA_021488915.1	clinical	2017	Homo sapiens	feces
GCA_021486075.1	clinical	2017	Homo sapiens	feces
GCA_021493485.1	clinical	2017	Homo sapiens	feces
GCA_021486055.1	clinical	2017	Homo sapiens	feces
GCA_021486255.1	clinical	2017	Homo sapiens	feces
GCA_021488755.1	clinical	2017	Homo sapiens	feces
GCA_021490975.1	clinical	2017	Homo sapiens	feces
GCA_021488835.1	clinical	2017	Homo sapiens	feces
GCA_021470125.1	clinical	2017	Homo sapiens	feces
GCA_021486335.1	clinical	2017	Homo sapiens	feces
GCA_021485175.1	clinical	2017	Homo sapiens	feces
GCA_021473865.1	clinical	2017	Homo sapiens	feces
GCA_021489255.1	clinical	2017	Homo sapiens	feces
GCA_021488735.1	clinical	2017	Homo sapiens	feces
GCA_021473845.1	clinical	2017	Homo sapiens	feces
GCA_021473825.1	clinical	2017	Homo sapiens	feces
GCA_021486035.1	clinical	2017	Homo sapiens	feces
GCA_021485155.1	clinical	2017	Homo sapiens	feces
GCA_021470105.1	clinical	2017	Homo sapiens	feces
GCA_021488715.1	clinical	2017	Homo sapiens	feces
GCA_021470085.1	clinical	2017	Homo sapiens	feces
GCA_021489235.1	clinical	2017	Homo sapiens	feces
GCA_021488675.1	clinical	2017	Homo sapiens	feces
GCA_021488695.1	clinical	2017	Homo sapiens	feces
GCA_021490715.1	clinical	2017	Homo sapiens	feces
GCA_021486015.1	clinical	2017	Homo sapiens	feces
GCA_021485975.1	clinical	2017	Homo sapiens	feces
GCA_021485935.1	clinical	2017	Homo sapiens	feces
GCA_021488845.1	clinical	2017	Homo sapiens	feces
GCA_021488655.1	clinical	2017	Homo sapiens	feces
GCA_021473805.1	clinical	2017	Homo sapiens	feces
GCA_021485135.1	clinical	2017	Homo sapiens	feces
GCA_021487475.1	clinical	2017	Homo sapiens	feces
GCA_021485195.1	clinical	2017	Homo sapiens	feces
GCA_021488815.1	clinical	2017	Homo sapiens	feces
GCA_021485535.1	clinical	2017	Homo sapiens	feces
GCA_021487655.1	clinical	2017	Homo sapiens	feces
GCA_021470145.1	clinical	2017	Homo sapiens	feces
GCA_021470065.1	clinical	2017	Homo sapiens	feces
GCA_021485995.1	clinical	2017	Homo sapiens	feces
GCA_021489175.1	clinical	2017	Homo sapiens	feces
GCA_021473765.1	clinical	2017	Homo sapiens	feces
GCA_021485515.1	clinical	2017	Homo sapiens	feces
GCA_021470045.1	clinical	2017	Homo sapiens	feces
GCA_021485215.1	clinical	2017	Homo sapiens	feces
GCA_021485495.1	clinical	2017	Homo sapiens	feces
GCA_021485955.1	clinical	2017	Homo sapiens	feces
GCA_021485095.1	clinical	2017	Homo sapiens	feces
GCA_021485075.1	clinical	2017	Homo sapiens	feces
GCA_021485105.1	clinical	2017	Homo sapiens	feces
GCA_021485475.1	clinical	2017	Homo sapiens	feces

GCA_021485455.1	clinical	2017	Homo sapiens	feces
GCA_021485055.1	clinical	2017	Homo sapiens	feces
GCA_021493465.1	clinical	2017	Homo sapiens	feces
GCA_021485435.1	clinical	2017	Homo sapiens	feces
GCA_021489215.1	clinical	2017	Homo sapiens	feces
GCA_021485415.1	clinical	2017	Homo sapiens	feces
GCA_021485375.1	clinical	2017	Homo sapiens	feces
GCA_021490075.1	clinical	2017	Homo sapiens	feces
GCA_021473705.1	clinical	2017	Homo sapiens	feces
GCA_021488795.1	clinical	2017	Homo sapiens	feces
GCA_021485395.1	clinical	2017	Homo sapiens	feces
GCA_021488775.1	clinical	2017	Homo sapiens	feces
GCA_021490635.1	clinical	2017	Homo sapiens	feces
GCA_021486615.1	clinical	2017	Homo sapiens	feces
GCA_021485915.1	clinical	2017	Homo sapiens	feces
GCA_021485355.1	clinical	2017	Homo sapiens	feces
GCA_021473905.1	clinical	2017	Homo sapiens	feces
GCA_021473885.1	clinical	2017	Homo sapiens	feces
GCA_021485335.1	clinical	2017	Homo sapiens	feces
GCA_021486295.1	clinical	2017	Homo sapiens	feces
GCA_021473785.1	clinical	2017	Homo sapiens	feces
GCA_021493445.1	clinical	2017	Homo sapiens	feces
GCA_021490615.1	clinical	2017	Homo sapiens	feces
GCA_021485255.1	clinical	2017	Homo sapiens	feces
GCA_021488895.1	clinical	2017	Homo sapiens	feces
GCA_021485895.1	clinical	2017	Homo sapiens	feces
GCA_021486555.1	clinical	2017	Homo sapiens	feces
GCA_021485315.1	clinical	2017	Homo sapiens	feces
GCA_021473725.1	clinical	2017	Homo sapiens	feces
GCA_021473685.1	clinical	2017	Homo sapiens	feces
GCA_021485855.1	clinical	2017	Homo sapiens	feces
GCA_021489195.1	clinical	2017	Homo sapiens	feces
GCA_021485835.1	clinical	2017	Homo sapiens	feces
GCA_021485295.1	clinical	2017	Homo sapiens	feces
GCA_021470745.1	clinical	2017	Homo sapiens	feces
GCA_021486575.1	clinical	2017	Homo sapiens	feces
GCA_021490555.1	clinical	2017	Homo sapiens	feces
GCA_021486515.1	clinical	2017	Homo sapiens	feces
GCA_021470165.1	clinical	2017	Homo sapiens	feces
GCA_021485275.1	clinical	2017	Homo sapiens	feces
GCA_021474125.1	clinical	2017	Homo sapiens	feces
GCA_021485235.1	clinical	2017	Homo sapiens	feces
GCA_025421815.1	clinical	2021	Homo sapiens	fecal samples
GCA_025421855.1	clinical	2021	Homo sapiens	fecal samples
GCA_025421865.1	clinical	2021	Homo sapiens	fecal samples
GCA_025421895.1	clinical	2021	Homo sapiens	fecal samples
GCA_025421915.1	clinical	2021	Homo sapiens	fecal samples
GCA_025421935.1	clinical	2021	Homo sapiens	fecal samples
GCA_025421955.1	clinical	2021	Homo sapiens	fecal samples
GCA_025421975.1	clinical	2021	Homo sapiens	fecal samples
GCA_025421985.1	clinical	2021	Homo sapiens	fecal samples
GCA_025422015.1	clinical	2021	Homo sapiens	fecal samples
GCA_025422045.1	clinical	2021	Homo sapiens	fecal samples
GCA_025422065.1	clinical	2021	Homo sapiens	fecal samples
GCA_025643135.1	animal breeding area	2017	swine	pharyngeal
GCA_025826075.1	clinical	2019	Homo sapiens	urine

GCA_025826055.1	clinical	2019	Homo sapiens	urine
GCA_025826515.1	clinical	2018	Homo sapiens	urine
GCA_025826495.1	clinical	2018	Homo sapiens	urine
GCA_025826775.1	clinical	2018	Homo sapiens	urine
GCA_025826035.1	clinical	2018	Homo sapiens	urine
GCA_025826015.1	clinical	2017	Homo sapiens	urine
GCA_025825995.1	clinical	2020	Homo sapiens	urine
GCA_025825935.1	clinical	2019	Homo sapiens	urine
GCA_025826795.1	clinical	2019	Homo sapiens	urine
GCA_025825955.1	clinical	2019	Homo sapiens	urine
GCA_025826755.1	clinical	2019	Homo sapiens	urine
GCA_025826355.1	clinical	2017	Homo sapiens	urine
GCA_025825895.1	clinical	2017	Homo sapiens	urine
GCA_025792805.1	clinical	2015	Homo sapiens	hospital
GCA_026247825.1	clinical	2022	Homo sapiens	hospital
GCA_026247845.1	clinical	2022	Homo sapiens	hospital
GCA_026247865.1	clinical	2022	Homo sapiens	hospital
GCA_026614445.1	animal breeding area	2016	environmental	chicken dung channel
GCA_026614435.1	animal breeding area	2016	environmental	chicken dung channel
GCA_026614515.1	animal breeding area	2016	chicken	chicken manure
GCA_026614545.1	animal breeding area	2016	chicken	chicken manure
GCA_026614595.1	animal breeding area	2016	chicken	chicken manure
GCA_026614615.1	animal breeding area	2016	chicken	chicken manure
GCA_026614645.1	animal breeding area	2016	pig	sick pig liver
GCA_026614655.1	animal breeding area	2016	chicken	chicken manure
GCA_026614695.1	animal breeding area	2016	environmental	chicken slaughterhouse splitting skin
GCA_026614715.1	animal breeding area	2016	chicken	chicken manure
GCA_026614735.1	animal breeding area	2016	chicken	chicken manure
GCA_026614745.1	animal breeding area	2016	chicken	chicken manure
GCA_026614775.1	animal breeding area	2016	chicken	chicken manure
GCA_026614785.1	animal breeding area	2016	chicken	chicken manure
GCA_026614815.1	animal breeding area	2016	chicken	chicken manure
GCA_026614825.1	animal breeding area	2016	chicken	chicken manure
GCA_026614855.1	animal breeding area	2016	chicken	sick chicken lung
GCA_026614875.1	animal breeding area	2016	chicken	sick chicken lung
GCA_026614895.1	animal breeding area	2016	pig	sick pig lung
GCA_026614975.1	animal breeding area	2016	chicken	sick chicken liver
GCA_026615015.1	clinical	2016	Homo sapiens	urine
GCA_026615055.1	clinical	2015	Homo sapiens	sputum
GCA_026615375.1	clinical	2015	Homo sapiens	sputum
GCA_026615405.1	clinical	2015	Homo sapiens	Catheter
GCA_026615485.1	clinical	2014	Homo sapiens	Pus
GCA_026615515.1	clinical	2015	Homo sapiens	pus
GCA_026615555.1	clinical	2015	Homo sapiens	Broncho-alveolar lavage
GCA_026615565.1	clinical	2015	Homo sapiens	urine
GCA_026615615.1	clinical	2016	Homo sapiens	sputum
GCA_026615795.1	clinical	2015	Homo sapiens	urine
GCA_026619475.1	clinical	2015	Homo sapiens	sputum
GCA_026619615.1	clinical	2016	Homo sapiens	urine
GCA_026619835.1	clinical	2016	Homo sapiens	urine
GCA_026619895.1	clinical	2016	Homo sapiens	pus
GCA_026619915.1	clinical	2016	Homo sapiens	urine
GCA_026619975.1	clinical	2016	Homo sapiens	sputum
GCA_028830215.1	animal breeding area	2019	Pig	pork
GCA_028830425.1	animal breeding area	2019	Pig	pork
GCA_028830155.1	animal breeding area	2019	Pig	pork

GCA_028829915.1	animal breeding area	2019	Pig	pork
GCA_028830865.1	animal breeding area	2019	Pig	pork
GCA_033564035.1	clinical	2023	Homo sapiens	urine
GCA_034334925.2	clinical	2023	Homo sapiens	hospital
GCA_034335725.2	clinical	2023	Homo sapiens	hospital
GCA_034336645.2	clinical	2023	Homo sapiens	hospital
GCA_034337405.2	clinical	2023	Homo sapiens	hospital
GCA_034338325.2	clinical	2023	Homo sapiens	hospital
GCA_034339465.2	clinical	2023	Homo sapiens	hospital
GCA_035819215.1	animal breeding area	2020	chicken	feces
GCA_035819235.1	animal breeding area	2020	chicken	feces
GCA_035819255.1	animal breeding area	2020	chicken	feces
GCA_035819275.1	animal breeding area	2020	chicken	feces
GCA_035819295.1	animal breeding area	2020	chicken	feces
GCA_035819315.1	animal breeding area	2020	chicken	feces
GCA_035819335.1	animal breeding area	2020	chicken	feces
GCA_035819375.1	animal breeding area	2020	chicken	feces
GCA_035819395.1	animal breeding area	2020	chicken	feces
GCA_035819415.1	animal breeding area	2020	chicken	feces
GCA_035826735.1	animal breeding area	2014	chicken	feces
GCA_035826755.1	animal breeding area	2014	chicken	feces
GCA_035827055.1	animal breeding area	2014	chicken	feces
GCA_035827075.1	animal breeding area	2014	chicken	feces
GCA_035827115.1	animal breeding area	2014	chicken	feces
GCA_035827195.1	animal breeding area	2014	chicken	feces
GCA_035827205.1	animal breeding area	2014	chicken	feces
GCA_035827275.1	animal breeding area	2014	chicken	feces
GCA_035967015.1	animal breeding area	2023	pigeon	dung
GCA_035967205.1	animal breeding area	2023	pigeon	dung
GCA_035967125.1	animal breeding area	2023	pigeon	dung
GCA_040537825.1	clinical	2024	Homo sapiens	hospital
GCA_040743225.1	clinical	2023	Homo sapiens	feces
GCA_040743235.1	clinical	2023	Homo sapiens	feces
GCA_041434975.1	clinical	2018	Homo sapiens	blood
GCA_025422035.1	clinical	2021	Homo sapiens	fecal samples
