# Supplementary figures and images for: Genetic Characterization, Transmission Pattern and Health Risk Analysis of Intestinal Colonization ESBL-Producing Escherichia coli in Vegetable Farming Population
Source: Microorganisms. 2024 Dec 20;12(12):2646. doi: 10.3390/microorganisms12122646 (PMC11727906; doi:10.3390/microorganisms12122646)

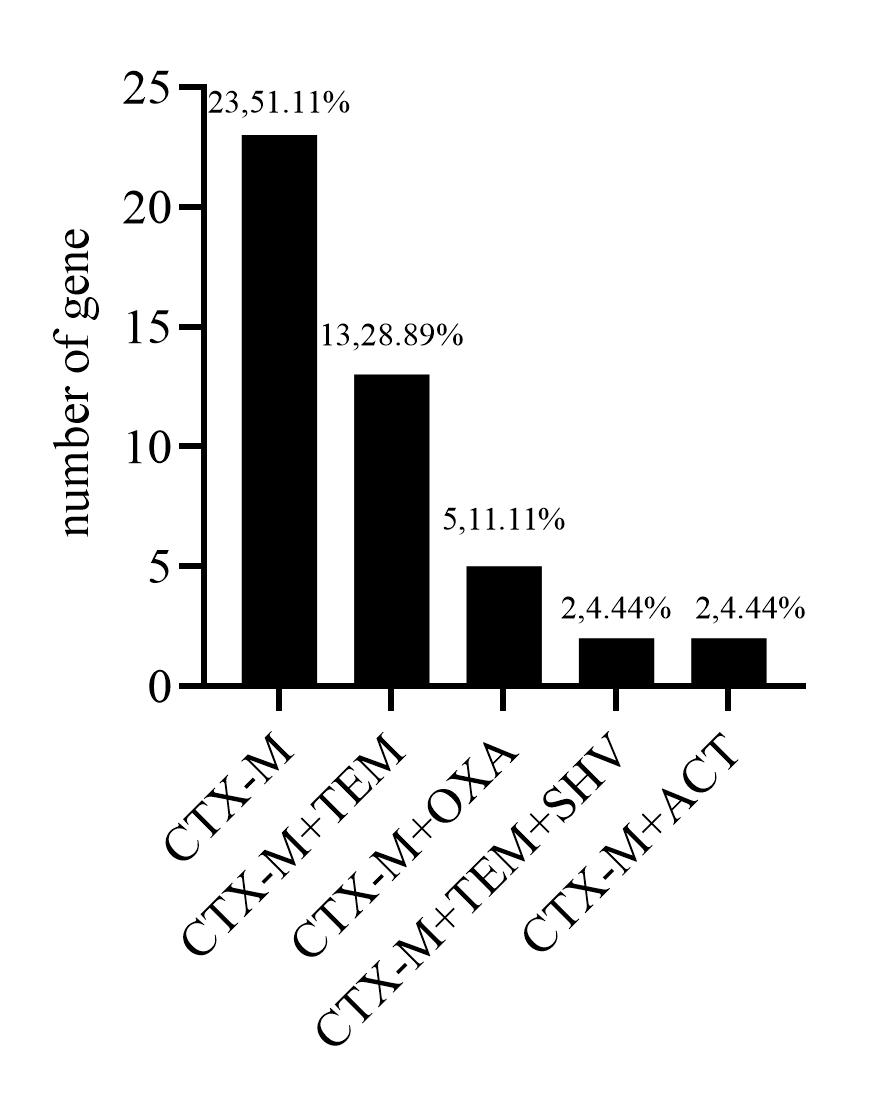

Supplement: Supplementary file 1 [file microorganisms-12-02646-s001.zip › Supplementary materials/Figure S1.jpg]

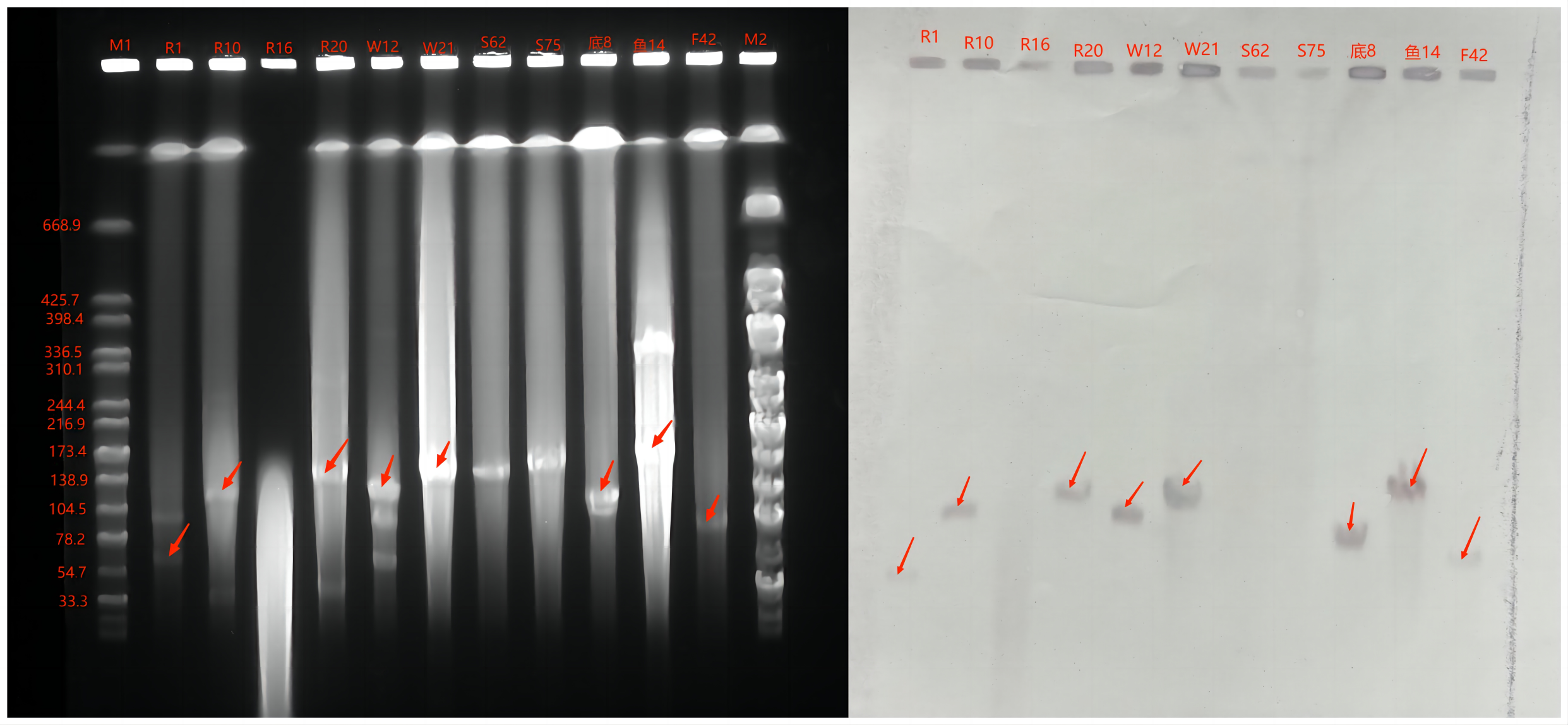

Supplement: Supplementary file 1 [file microorganisms-12-02646-s001.zip › Supplementary materials/Figure S2(A).png]

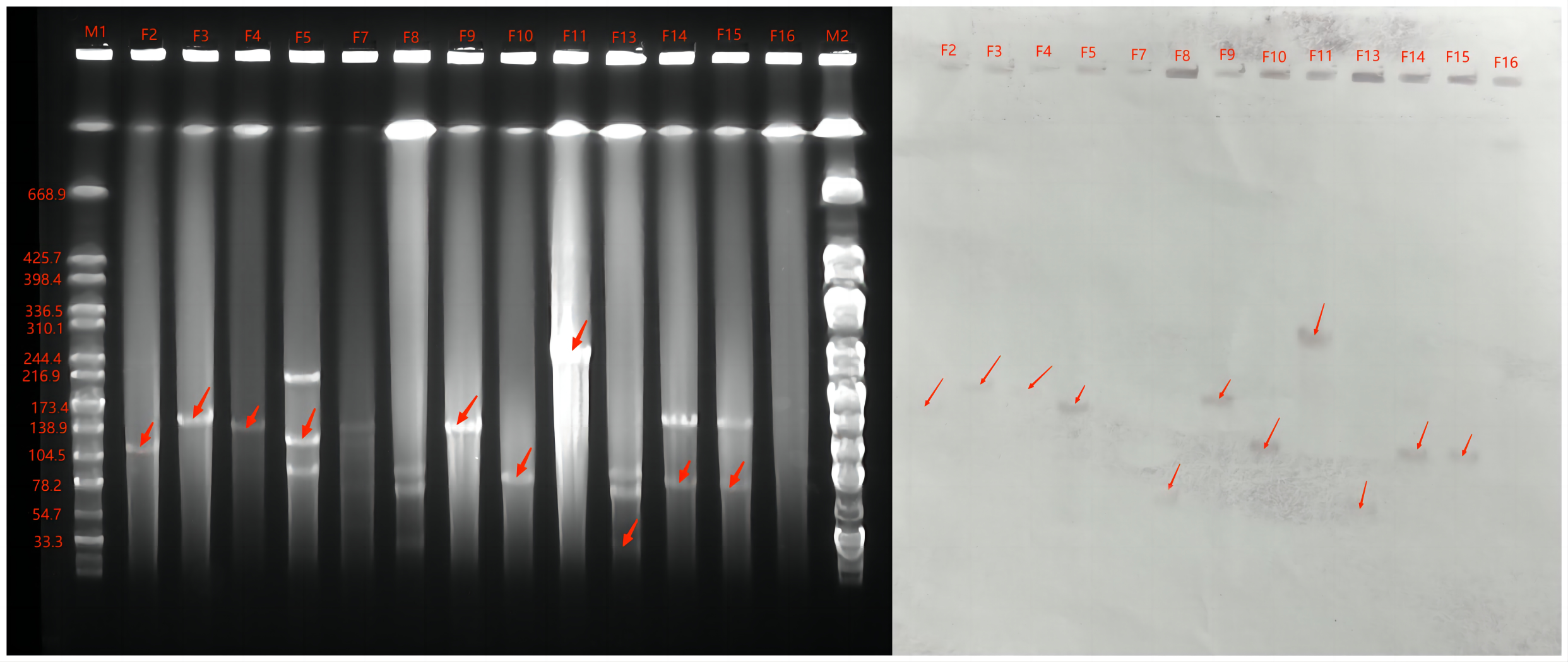

Supplement: Supplementary file 1 [file microorganisms-12-02646-s001.zip › Supplementary materials/Figure S2(B).png]

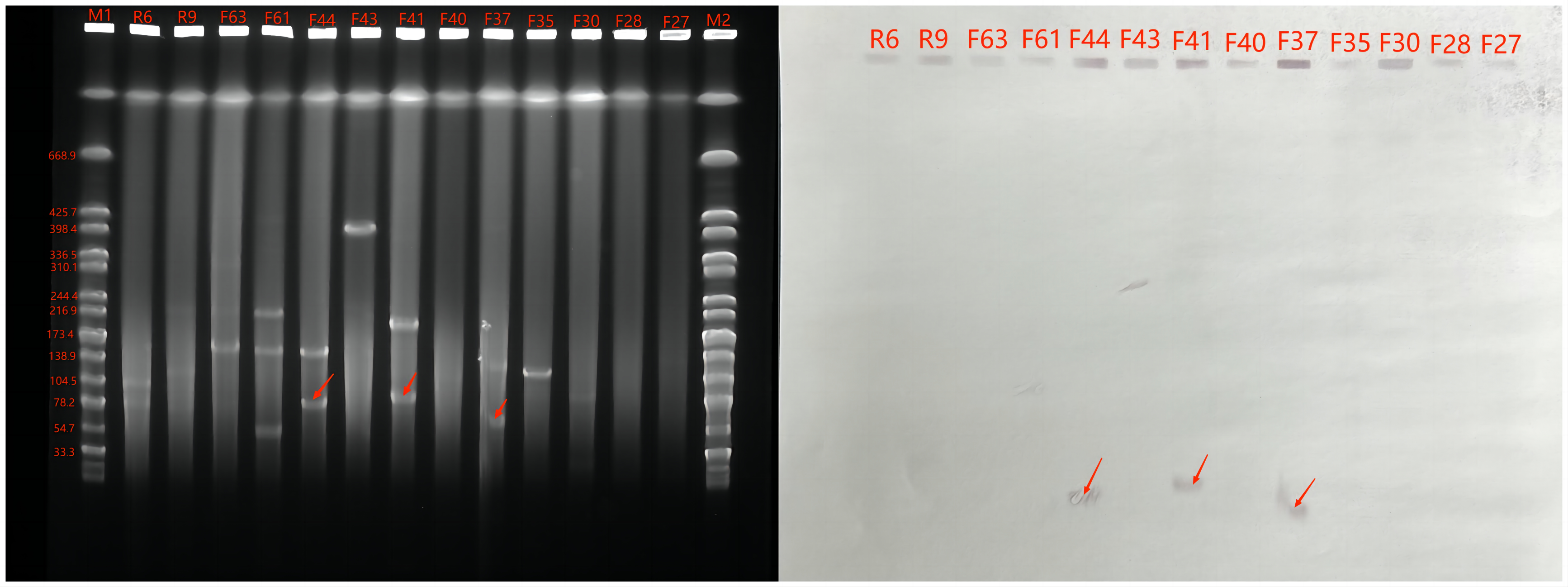

Supplement: Supplementary file 1 [file microorganisms-12-02646-s001.zip › Supplementary materials/Figure S2(C).png]

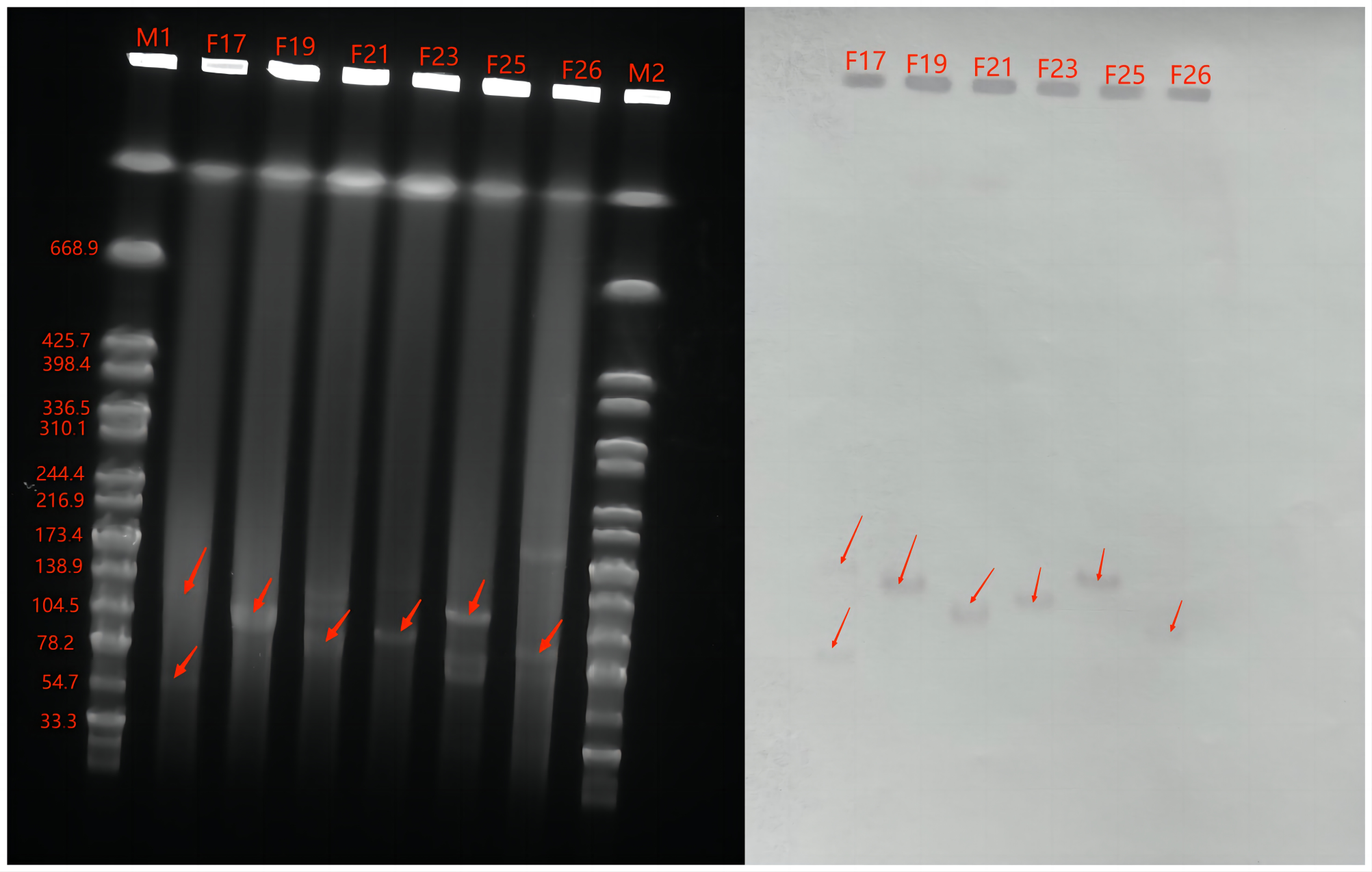

Supplement: Supplementary file 1 [file microorganisms-12-02646-s001.zip › Supplementary materials/Figure S2(D).png]

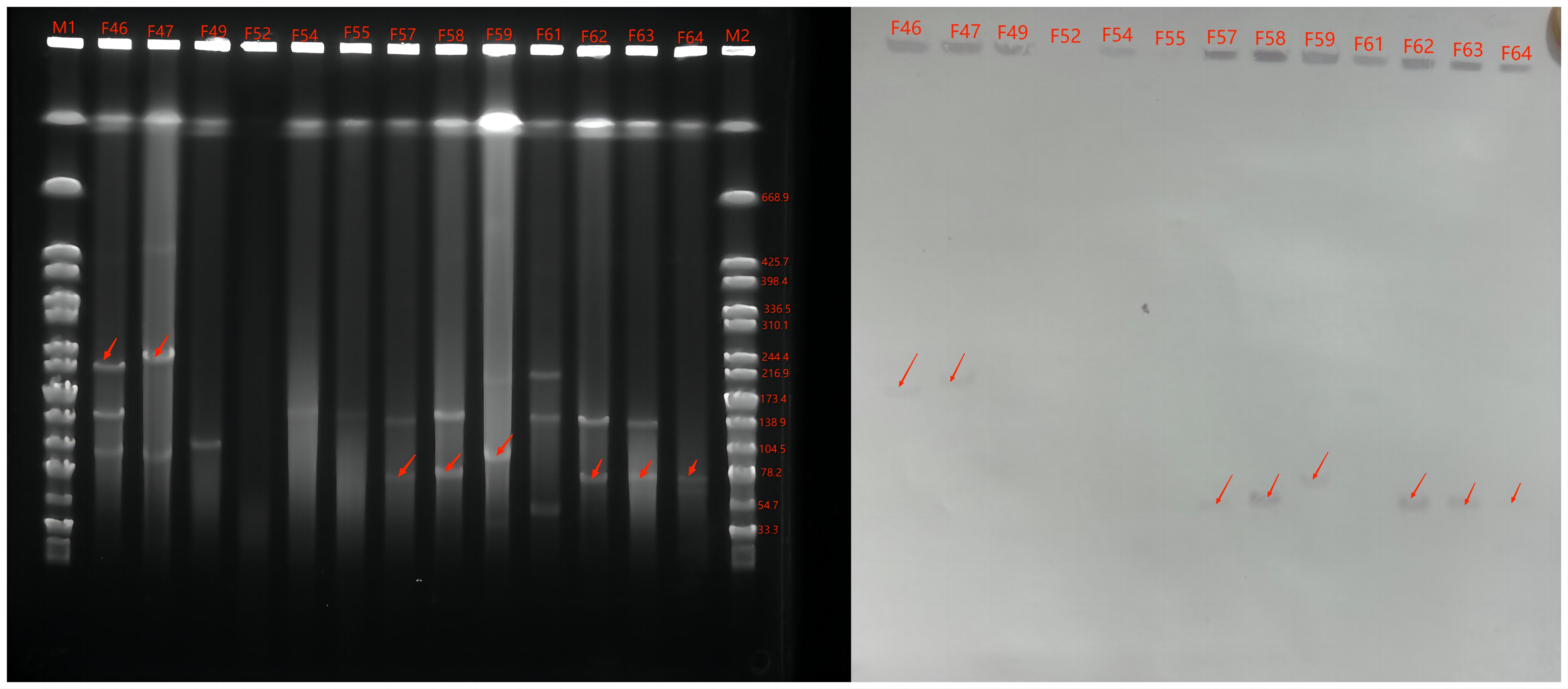

Supplement: Supplementary file 1 [file microorganisms-12-02646-s001.zip › Supplementary materials/Figure S2(E).png]

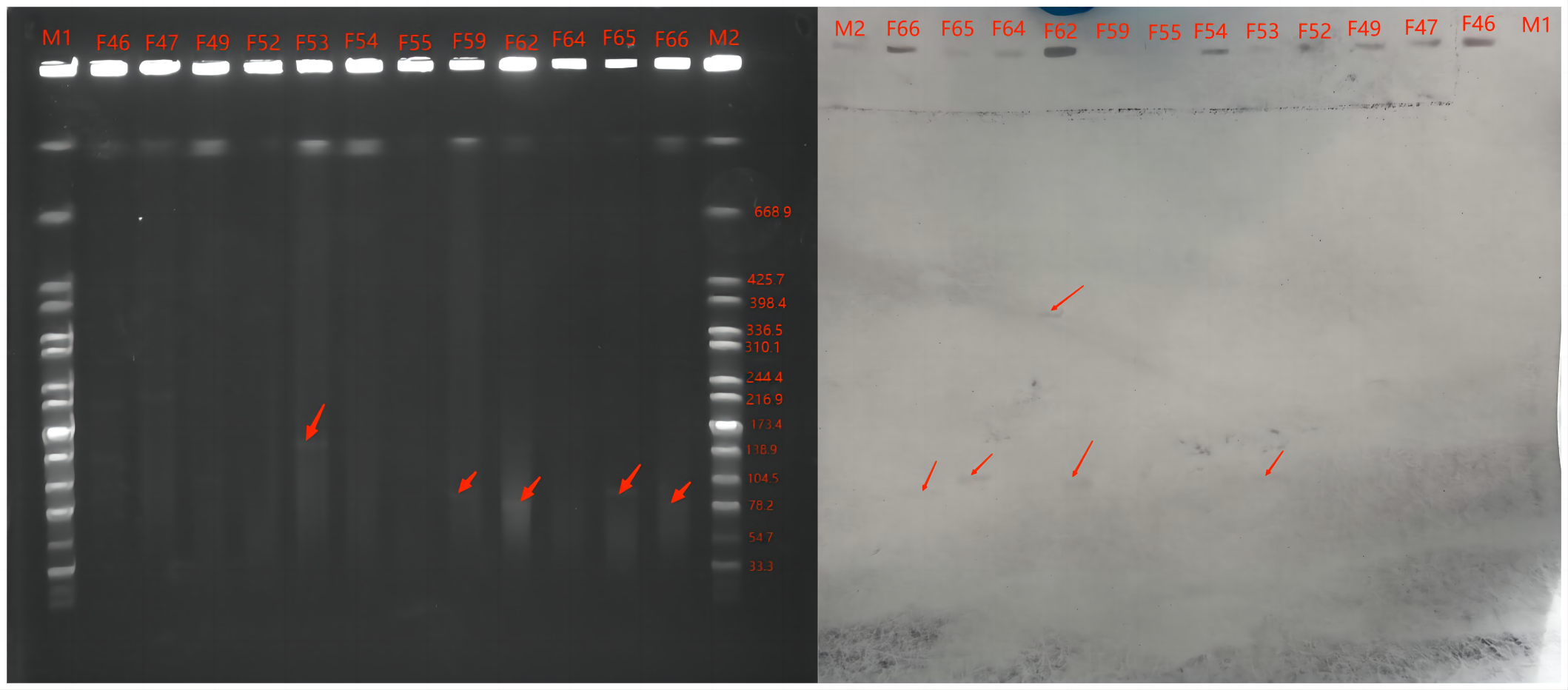

Supplement: Supplementary file 1 [file microorganisms-12-02646-s001.zip › Supplementary materials/Figure S2(F).png]

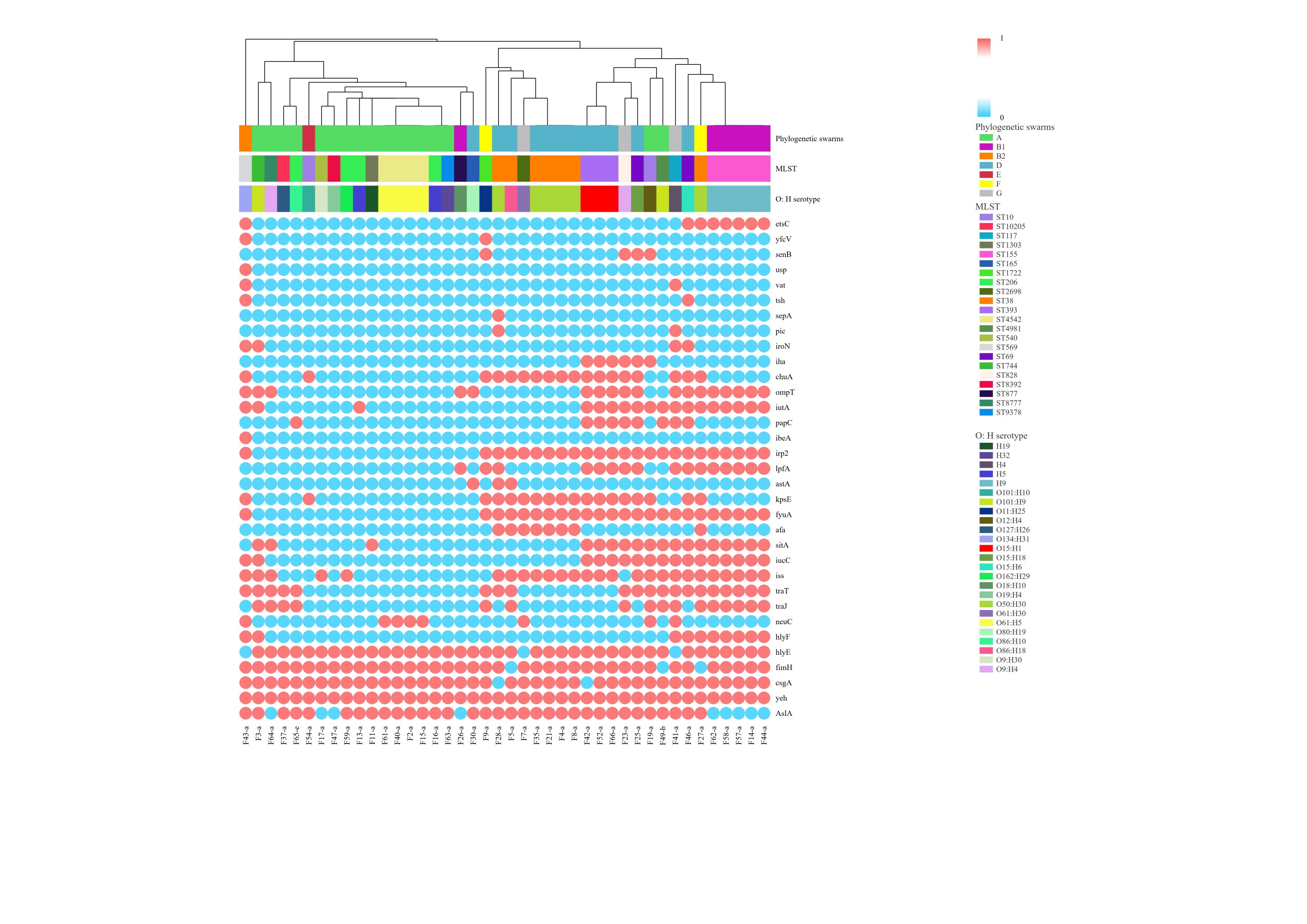

Supplement: Supplementary file 1 [file microorganisms-12-02646-s001.zip › Supplementary materials/Figure S3.jpg]
